# *p*HBMT1, a BAHD-family monolignol acyltransferase, mediates lignin acylation in poplar

**DOI:** 10.1093/plphys/kiab546

**Published:** 2021-11-22

**Authors:** Lisanne de Vries, Heather A MacKay, Rebecca A Smith, Yaseen Mottiar, Steven D Karlen, Faride Unda, Emilia Muirragui, Craig Bingman, Kirk Vander Meulen, Emily T Beebe, Brian G Fox, John Ralph, Shawn D Mansfield

**Affiliations:** 1 Department of Wood Science, Faculty of Forestry, University of British Columbia, Vancouver, BC V6T 1Z4, Canada; 2 US Department of Energy (DOE) Great Lakes Bioenergy Research Center, the Wisconsin Energy Institute, University of Wisconsin-Madison, Madison, Wisconsin 53726, USA; 3 Department of Biochemistry, University of Wisconsin-Madison, Madison, Wisconsin 53706, USA

## Abstract

Poplar (*Populus*) lignin is naturally acylated with *p*-hydroxybenzoate ester moieties. However, the enzyme(s) involved in the biosynthesis of the monolignol–*p*-hydroxybenzoates have remained largely unknown. Here, we performed an *in vitro* screen of the *Populus trichocarpa* BAHD acyltransferase superfamily (116 genes) using a wheatgerm cell-free translation system and found five enzymes capable of producing monolignol–*p*-hydroxybenzoates. We then compared the transcript abundance of the five corresponding genes with *p*-hydroxybenzoate concentrations using naturally occurring unrelated genotypes of *P. trichocarpa* and revealed a positive correlation between the expression of *p*-hydroxybenzoyl-CoA monolig-nol transferase (*pHBMT1*, Potri.001G448000) and *p*-hydroxybenzoate levels. To test whether *pHBMT1* is responsible for the biosynthesis of monolignol–*p*-hydroxybenzoates, we overexpressed *pHBMT1* in hybrid poplar (*Populus alba* × *P. grandidentata*) (*35S::pHBMT1* and *C4H::pHBMT1*). Using three complementary analytical methods, we showed that there was an increase in soluble monolignol–*p*-hydroxybenzoates and cell-wall-bound monolignol–*p*-hydroxybenzoates in the poplar transgenics. As these pendent groups are ester-linked, saponification releases *p*-hydroxybenzoate, a precursor to parabens that are used in pharmaceuticals and cosmetics. This identified gene could therefore be used to engineer lignocellulosic biomass with increased value for emerging biorefinery strategies.

## Teaser

An enzyme from poplar, *p*-hydroxybenzoyl-CoA monolignol transferase (*pHBMT1*), adds ester-linked *p*-hydroxybenzoate moieties to lignin that can be readily hydrolyzed to release the aromatic compound.

## Introduction

Cellulose, hemicelluloses, and the phenolic polymer lignin are the three main components of the secondary cell walls of vascular plants. Lignin is crucial as it protects plants against herbivores and pathogens, and provides strength to stems and vascular tissues thereby facilitating upward growth and long-distance water transport (Weng and Chapple, 2010; Miedes et al., 2014). Lignin is synthesized in the apoplast via oxidative coupling of monolignols (primarily, *p*-coumaryl alcohol, coniferyl alcohol, and sinapyl alcohol) that are produced in the cytosol via the phenylpropanoid pathway (Boerjan et al., 2003). Given that oxidative coupling is a purely chemical process (Freudenberg and Neish, 1968; Ralph et al., 2008), a series of noncanonical lignin monomers, which may be naturally occurring or engineered, can be incorporated into lignin (Mottiar et al., 2016; Vanholme et al., 2019). Moreover, the list of noncanonical monomers that have been identified continues to grow, and includes the flavonoids naringenin and tricin, curcumin, caffeyl alcohol, and hydroxystilbenes, such as resveratrol, piceatannol, and isorhapontigenin (Chen et al., 2012; Lan et al., 2016; del Río et al., 2017; Lam et al., 2017; Oyarce et al., 2019; Mahon et al., 2021). These noncanonical monomers can alter the physicochemical properties of lignin and/or affect its interactions with other cell wall components, thus rendering the lignocellulosic biomass less recalcitrant to chemical deconstruction. In addition, the molecules themselves are high-value compounds or can be employed as useful chemical precursors (de Vries et al., 2021).

Another group of noncanonical monomers is the acylated (ester-linked) monolignols. The classical example of these is the monolignol–*p*-coumarates that are found in the cell walls of commelinid monocots (Ralph et al., 1994; Karlen et al., 2018). Monolignol–*p*-coumarates are synthesized by *p*-coumaroyl-CoA monolignol transferases (PMTs), members of the BAHD enzyme family that are responsible for conjugating *p*-coumaroyl-CoA with monolignols (Hatfield et al., 2009; Withers et al., 2012; Marita et al., 2014; Petrik et al., 2014; Smith et al., 2015; Karlen et al., 2018). The *p*-coumarate moieties of these conjugates preferentially transfer radicals rather than undergo radical coupling reactions, resulting in a lignin decorated with free-phenolic *p*-coumarate pendent groups that are linked to the polymer via ester bonds (Hatfield et al., 2008; Ralph, 2010).

**Figure 1:**
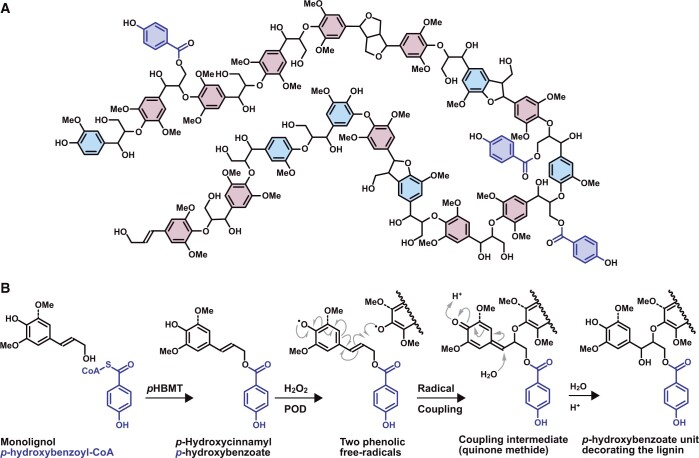
Model of a *p*-hydroxybenzoylated lignin, and scheme showing how *p*HB enters lignification. (A) Model of a typical poplar lignin, showing *p*HB esters (dark blue) acylating primary hydroxyl groups on the lignin polymer. (B) Biosynthesis of monolignol–*p*HB conjugates and their incorporation into the lignin polymer. *p*-Hydroxybenzoyl-CoA and *p*HB are in dark blue.

Naturally found in poplar/aspen (*Populus*) and willow (*Salix*), another group of acylated monolignols are the monolignol–*p*-hydroxybenzoates (henceforth denoted as *p*HB to indicate the ester-linked form; Smith, 1955; Ralph et al., 2004; Lu et al., 2015). As with monolignol–*p*-coumarates, the *p*-hydroxybenzoyl moieties of such conjugates undergo radical transfer, resulting in lignin that is decorated with *p*HB pendent groups (Figure 1A; Lu et al., 2004; Morreel et al., 2004; Ralph, 2010). In poplar, it has been observed that *p*HB is almost exclusively bound to S-lignin units in the lignin of xylem fibers (Stewart et al., 2009; Regner et al., 2018; Goacher et al., 2021). As these moieties are linked to lignin via ester bonds, mild alkaline hydrolysis (saponification) releases these groups, which are therefore known as “clip-offs” (Rinaldi et al., 2016). These groups could theoretically be separated and used as high-value phenolic chemicals. For example, *p*-hydroxybenzoic acid (*p*HBA, indicating the “free” form) can be esterified with various alcohols to produce parabens that are commonly used as preservatives in the pharmaceutical and cosmetics industries (Yang et al., 2018).

To date, the biosynthesis of monolignol–*p*HB conjugates has remained largely unknown. In this study, we describe a *p*-hydroxybenzoyl-CoA monolignol transferase (*pHBMT*), a member of the BAHD acyltransferase gene family in poplar, that is responsible for the formation of monolignol-*p*HB conjugates (Figure 1B). To test whether *pHBMT* is ultimately responsible for *p*-hydroxybenzoylation of poplar lignin, we overexpressed this gene in hybrid poplar. This resulted in an increased level of *p*HB groups in the cell wall, but had no effect on the biomass content or lignin concentration, suggesting that this may be a promising strategy to enhance biomass value for the biorefinery.

## Results

### Identification of putative *p*HBMTs from *Populus trichocarpa*

By analogy with the known PMTs and feruloyl-CoA monolignol transferases (FMTs; Withers et al., 2012; Petrik et al., 2014; Marita et al., 2014; Wilkerson et al., 2014; Karlen et al., 2016), *p*HBMTs were expected to be part of the Pfam 02458 family of CoA-dependent transferases (BAHD enzyme family). To identify putative *p*HBMTs that are capable of acylating monolignols with *p*HBA, the *P. trichocarpa* protein sequences with substantial matches to the hidden Markov model (HMM) profile for the BAHD enzyme family (Pfam model PF02458; Tuominen et al., 2011), were retrieved from Phytozome, which yielded 147 candidates. Of these, 20 sequences were too short (<232 amino acids) to form a complete PF02458 protein, and 6 sequences were nearly identical to one another, and therefore only one of the pair was synthesized (see details in Supplemental Data Set S1). Consequently, this yielded putative BAHD acyltransferases that were organized into clades based on the phylogenetic relatedness of their amino acid sequences (Supplemental Figure S1).

**Figure 2:**
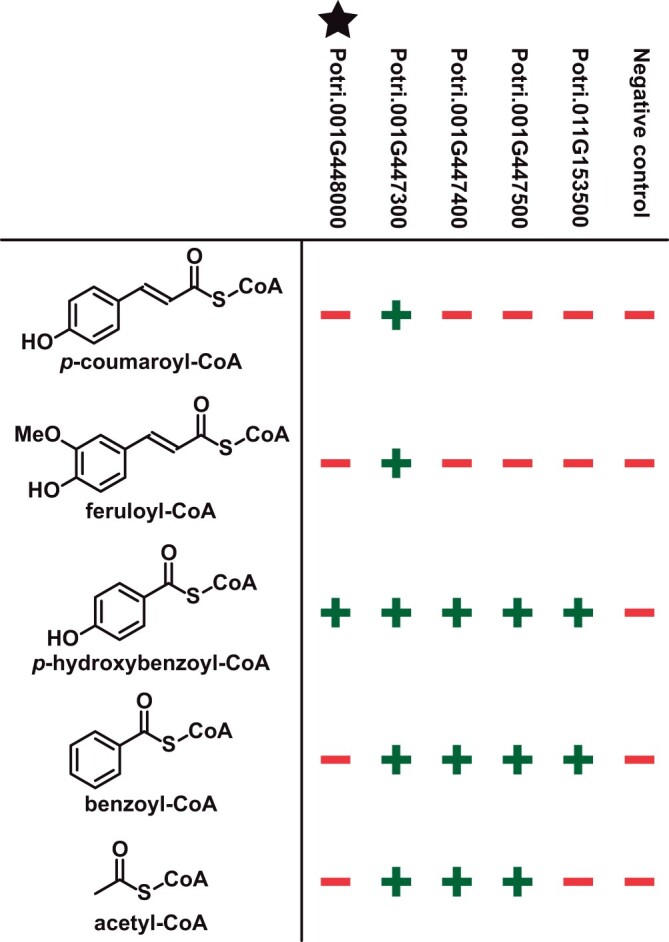
Activity of putative *p*HBMT enzymes towards different CoA donors and monolignol acceptors. For each enzyme, the five different CoA donors were simultaneously tested with three canonical monolignol acceptors (*p*-coumaryl alcohol, coniferyl alcohol, and sinapyl alcohol) in the multiplexed assay. A “+” indicates the formation of detectable levels of monolignol conjugate products when the respective enzyme was added. A “−” indicates that no detectable monolignol conjugate products were formed in the presence of the respective enzyme. The negative control consisted of a blank wheat germ translation reaction supplemented with the indicated CoA donor and the three monolignol acceptors.

Among the 121 genes submitted to the Joint Genome Institute, 116 BAHD acyltransferase genes were successfully synthesized and incorporated into a plasmid used for wheatgerm cell-free protein synthesis. All individual plasmids were used to carry out cell-free protein translation, and the translation reactions were pooled into groups of 10 and screened for activity against 5 acyl-donor substrates (*p*-coumaroyl-CoA, feruloyl-CoA, *p*-hydroxybenzoyl-CoA, benzoyl-CoA, and acetyl-CoA) with the three monolignol acyl acceptors (*p*-coumaryl alcohol, coniferyl alcohol, and sinapyl alcohol). Pools of enzymes with a positive hit for activity with one or more of the donors were then re-examined in individual enzyme assays. Of the 116 reactions tested, 7 showed reactivity with the substrates tested, and only 5 showed *in vitro* activity with *p*-hydroxybenzoyl-CoA, the presumed substrate for *p*HBMT enzymes (Figure 2). Moreover, of the 5 putative *p*HMBTs, only one showed activity exclusively with *p*-hydroxybenzoyl-CoA, whereas the others also displayed some activity *in vitro* toward acetyl-CoA, benzoyl-CoA, *p*-coumaroyl-CoA, and feruloyl-CoA (Figure 2). These 5 genes all clustered into a single clade (Supplemental Figure S1).

### Natural variation in *p*HB concentration in *P. trichocarpa*

**Figure 3:**
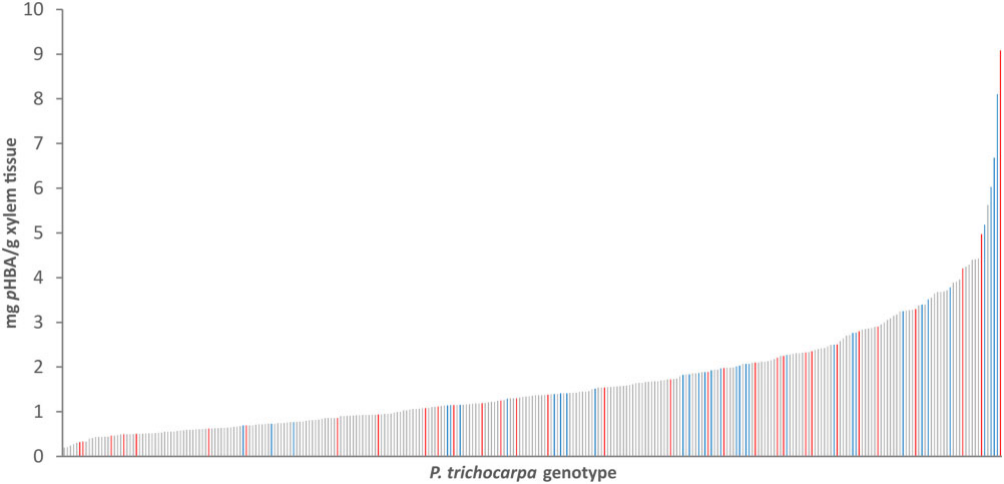
Cell-wall-bound *p*HB (expressed as mg *p*HBA/g xylem tissue) of 316 9-year-old *P. trichocarpa* genotypes. The *p*HB amounts are ranked from low to high. Blue: *P. trichocarpa* genotypes originating from 44°N < latitude < 49.1°N, gray: *P. trichocarpa* genotypes originating from 49.12°N < latitude < 52.72°N, red: *P. trichocarpa* genotypes originating from 52.77°N < latitude < 54.18°N.

**Figure 4:**
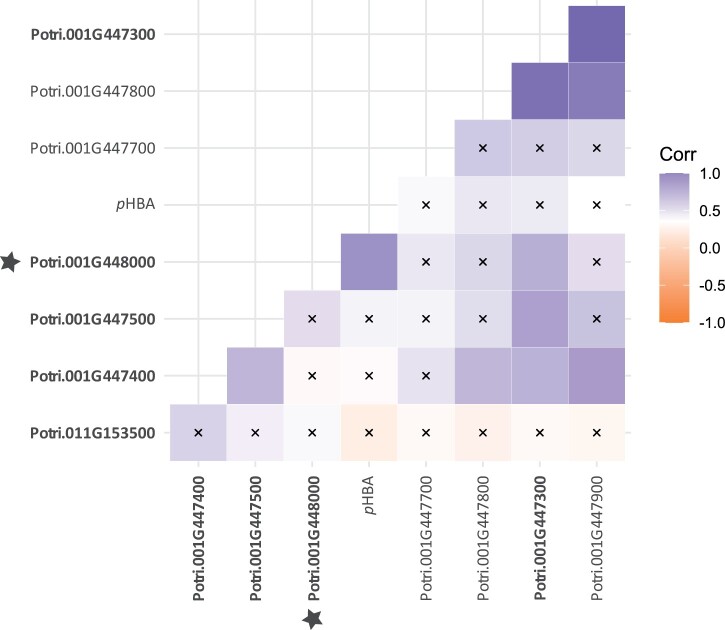
Spearman correlation matrix of xylem RNA expression levels of the BAHD clade containing the five putative *p*HBMTs and *p*HB content (expressed as mg *p*HBA/g xylem tissue). Potri.001G448000 (bold and star) is the only gene that shows significant correlation with *p*HB. Gene names in bold have *in vitro* activity towards *p*-hydroxybenzoyl-CoA, but do not show a significant correlation with *p*HB amount (except for Potri.001G448000). The other genes have no activity towards *p*-hydroxybenzoyl-CoA, but belong to the same phylogenetic clade (see Supplemental Figure S1). **“**x” marks correlations with *P* > 0.001389 (i.e., not statistically significant, Bonferroni correction).

The inherent xylem *p*HB concentration of 4-year-old field-grown *P. trichocarpa* trees representing 316 unrelated genotypes that span the natural range of the species was determined by high performance liquid chromatography (HPLC) following alkaline hydrolysis of extractive-free wood flour. The *p*HB content ranged from 0.20 mg *p*HB/g xylem tissue (genotype: KLND20-2) to 9.1 mg *p*HB/g xylem tissue (genotype: QLKE16-3; Figure 3). We performed a Spearman correlation analysis with the determined *p*HB contents and the gene expression of the five putative *pHBMTs* from our in-house RNA-sequencing (RNA-seq) database of developing xylem tissue. This clearly showed that only the expression of Potri.001G44800 had a significant positive correlation with *p*HB levels (Figure 4). And, in most poplar accessions, Potri.011G153500 and Potri.001G447400 did not show any expression in developing xylem whatsoever. Using the publicly available www.popgenie.org expression database (Sjödin et al., 2009; Sundell et al., 2015, 2017), Potri.001G448000 was the only one of the five candidates that displayed higher expression in *Populus* internode tissue compared to mature leaf tissue, and had the highest expression in developing xylem. Potri.001G448000 was also the most divergent from the other candidate genes (Supplemental Figure S1), and this gene was also co-expressed with several core lignin biosynthetic genes (Supplemental Figure S2 and Supplemental Table S1).

Taken together, Potri.001G448000 (hereafter denoted as *pHBMT1*) was identified as the most promising candidate, and was therefore selected for further *in vitro* enzyme activity assays and *in planta* analysis by overexpression of this gene in poplar.

### *In vitro* activity of *p*HBMT1

*p*HBMT1 was tested for activity with acetyl-CoA, *p*-coumaroyl-CoA, feruloyl-CoA, benzoyl-CoA, and *p*-hydroxybenzoyl-CoA as acyl donors and three monolignols as acyl acceptors (*p*-coumaryl alcohol, coniferyl alcohol, and sinapyl alcohol). The expected conjugate product was only observed in the reaction between monolignols and *p*-hydroxybenzoyl-CoA, based on liquid chromatography–mass spectrometry (LC–MS) analysis and comparison with authentic standards (Supplemental Figure S3 for spectra). When provided all three monolignols, *p*HBMT1 had a strong preference for sinapyl alcohol over coniferyl alcohol, and did not demonstrate any activity with *p*-coumaryl alcohol. Enzyme kinetics assays confirmed these observations (Table 1). The highest catalytic efficiency (*k*_cat_/*K_M_)* was evident with sinapyl alcohol and *p*HBA (saturated amount of *p*-hydroxybenzoyl-CoA and variable amounts of sinapyl alcohol). When comparing the kinetics of *p*HBMT1 to that of other known BAHD acyltransferases, no apparent trend was observed between *p*HBMT1, FMT, and PMT for example, which are all BAHD acyltransferases that use monolignols as substrate (Withers et al., 2012; Wilkerson et al., 2014).

**Table 1:** Michaelis–Menten enzyme kinetics parameters for *p*HBMT1. *V*_max_, *K*_M_, *k*_cat_, and *k*_cat_/*K*_M_ values with standard deviations for three technical replicates are shown for *p*-hydroxybenzoyl-CoA as an acyl acceptor and for sinapyl alcohol and coniferyl alcohol as acyl donors

Varying substrate	Saturating substrate	*V*_max_ (*p*_kat_/mg)	*K*_M_ (µM)	*k*_cat_ (s^-1^)	*k*_cat_/*K*_M_
*p*-Hydroxybenzoyl-CoA	Sinapyl alcohol	1714 ± 300	448 ± 21	0.18 ± 0.03	0.40 ± 0.07
Sinapyl alcohol	*p*-Hydroxybenzoyl-CoA	1284 ± 143	300 ± 72	0.13 ± 0.01	0.49 ± 0.09
*p*-Hydroxybenzoyl-CoA	Coniferyl alcohol	1298 ± 308	624 ± 195	0.14 ± 0.03	0.23 ± 0.02
Coniferyl alcohol	*p*-Hydroxybenzoyl-CoA	948 ± 211	954 ± 411	0.10 ± 0.02	0.13 ± 0.03

### Overexpression of *pHBMT1* in hybrid poplar does not influence plant biomass production

In order to test if *pHBMT1* expression can drive *p*HB production in plants, the synthetic gene (Potri.001G448000, codon-altered for gene synthesis) was expressed using two strong promoters, *35S::pHBMT1* and *C4H::pHBMT1*, in hybrid poplar (*Populus alba* × *P. grandidentata*; P39). For both transformations, 12 independent transformants were initially selected, and the three highest expressing lines (tissue-culture grown) for each construct were transferred to the greenhouse for growth and in-depth analysis.

**Figure 5:**
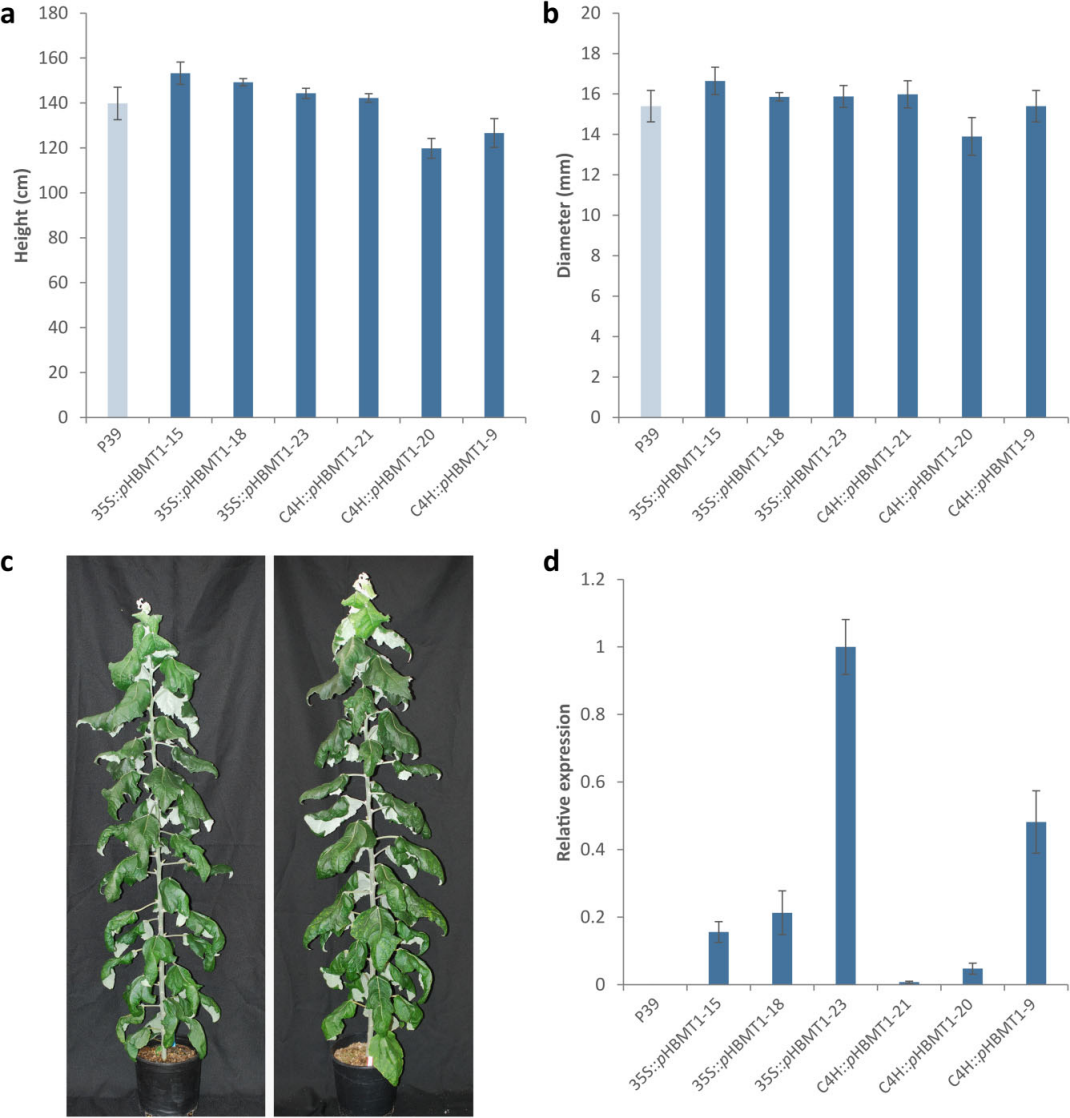
Height, diameter, and expression of *35S::pHBMT1* and *C4H::pHBMT1* poplars. (A) Height (cm) and (B) diameter (mm) of 4-month-old hybrid WT poplar (P39), *35S::pHBMT1*, and *C4H::pHBMT1* lines (*n* = 5 biological replicates for each line). (C) Representative 4-month-old WT line (left) and 4-month-old *35S::pHBMT1-23* line (right). (D) Expression analysis of *pHBMT1* in transgenic lines, normalized to the highest expressing line (*35S::pHBMT1-23*). *PtEF1*β was employed as a reference gene, *n *=* *3 biological replicates for each line (each with three technical replicates). All error bars represent sem.

An examination of height (cm) and diameter (mm) at the base of the stem just above the root collar following 4 months of growth did not reveal any alterations in the growth patterns of the transgenic lines compared to wild-type (WT) trees (Figure 5, A–C). The 4-month-old trees were then harvested and the expression of *pHBMT1* in developing xylem was confirmed using reverse transcription quantitative polymerase chain reaction (RT-qPCR) (Figure 5D).

### Increased amount of *p*HB in *p*HBMT1 lines

**Figure 6:**
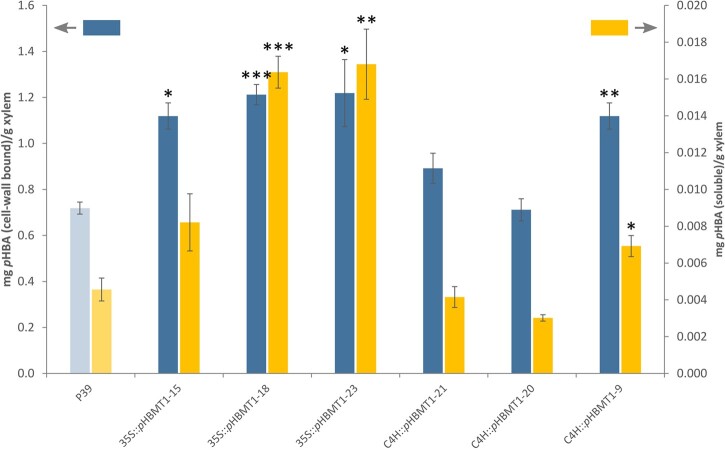
Cell-wall-bound *p*HB (expressed as mg *p*HBA/g xylem tissue post-saponification; left *y*-axis, blue) and soluble *p*HB (expressed as mg *p*HBA/g xylem tissue post-saponification; right *y*-axis, yellow) of WT (P39), *35S::pHBMT1*, and *C4H::pHBMT1* poplars. The methanol extract of 4-month-old xylem tissue was saponified and quantified via HPLC to determine the amount of soluble *p*HBA. The remaining cell wall fraction was also saponified and analyzed on a HPLC to determine the amount of cell-wall-bound *p*HB. *n *=* *3 biological replicates per line (each with two technical replicates), error bars represent sem. Statistical differences determined via Student’s *t*-test: *0.05 > *P *>* *0.01; **0.01 > *P *>* *0.001; and ***P <* *0.001.

**Figure 7:**
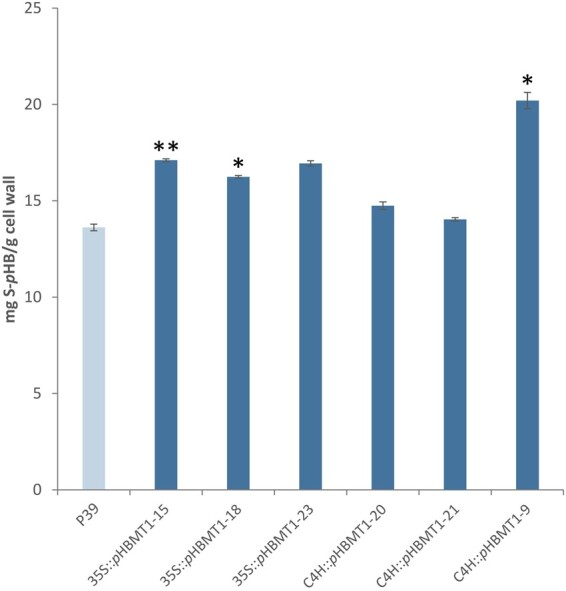
Amount of S-*p*HB released by DFRC of WT (P39), *35S::pHBMT1*, and *C4H::pHBMT1* poplars. Values are expressed in mg released S–*p*-hydroxybenzoate (S–*p*HB/g) cell wall. *n *=* *3 biological replicates per line (each with two technical replicates), error bars represent sem. Statistical differences determined via Student’s *t*-test: *0.05 > *P *>* *0.01.

**Figure 8:**
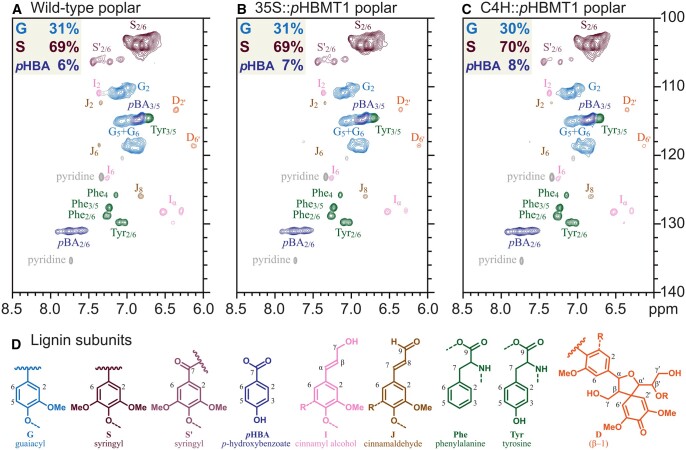
Lignin analysis by 2D-NMR. A–C, Representative heteronuclear single quantum coherence spectra showing the aromatic region of enzyme lignin of (A) WT poplar (P39), (B) *35S::pHBMT1*, and (C) *C4H::pHBMT1*. Integrated values of G, S, and *p*HBA units are given on an S + G = 100% basis. (D) Aromatic substructures colored to correspond with the signals in the heteronuclear single-quantum coherence spectra.

Following harvest, we determined the amount of *p*HB in the cell walls of the different transgenic poplar lines. Methanol extractions were performed on xylem tissue of each line and subsequently subjected to alkaline hydrolysis to determine the total amount of soluble *p*HBA (i.e., not bound to the cell wall). The remaining cell-wall residues were then acetone-washed and subjected to alkaline hydrolysis to determine the amount of cell-wall-bound *p*HB. HPLC analysis of these samples revealed that there was a substantial increase (80%–270%) in the soluble *p*HBA content, and also a significant increase (55%–70%) in cell-wall-bound *p*HB in all three *35S::pHBMT1* lines (Figure 6). In contrast, only the highest expressing *C4H::pHBMT1* line (line 9) displayed a significant increase in soluble and cell-wall-bound *p*HB (Figure 6). Derivatization followed by reductive cleavage (DFRC) was then used to determine the relative levels of monolignol–*p*HB conjugates released from lignin. As this method cleaves only β-ether bonds while leaving ester bonds intact (Lu and Ralph, 1999; Regner et al., 2018), DFRC can be used to quantify the relative amount of S-*p*HB incorporated into the lignin. As such, we observed a significant increase in the amount of released S-*p*HB for *35S::pHBMT1* line 15 and *35S::pHBMT1* line 18 (30% and 20% increase, respectively, Figure 7), and for *C4H::pHBMT1* line 9 (50% increase, Figure 7). Finally, nuclear magnetic resonance (NMR) was used to validate these findings and assess whether there were additional changes in lignin composition or structure. The relative intensity of the signal corresponding to *p*HB was increased in *35S::pHBMT1* line 15 (7.2%) and *C4H::pHBMT1* line 9 (7.9%) compared to its corresponding WT line (5.8%; Figure 8). No differences were observed in the S/G monomer ratio or in the proportions of interunit linkages (Table 2).

**Table 2:** Lignin characteristics of *35S::pHBMT1* and *C4H::pHBMT1* transgenic poplar trees

Line	S/G ratio (NMR)	%*p*HB (NMR)	%A	%B	%C	Acid-insoluble lignin (%CW)	Acid-soluble lignin (%CW)	Total lignin (%CW)
P39 (WT)	2.24 ± 0.02	5.8 ± 0.3	87.3 ± 0.1	5.3 ± 0.1	7.4 ± 0.1	16.69 ± 0.28	3.34 ± 0.17	20.02 ± 0.28
35S::*p*HBMT1-15	2.27 ± 0.03	**7.2 ± 0.4**	87.3 ± 0.4	5.5 ± 0.1	7.2 ± 0.5	16.70 ± 0.03	3.59 ± 0.09	20.29 ± 0.12
35S::*p*HBMT1-18	2.21 ± 0.03	**7.4 ± 0.3**	87.1 ± 0.1	5.7 ± 0.01	7.2 ± 0.1	17.17 ± 0.06	3.56 ± 0.14	20.74 ± 0.16
35S::*p*HBMT1-23	2.27 ± 0.03	7.3 ± 1.0	87.4 ± 0.1	5.2 ± 0.1	7.4 ± 0.1	16.50 ± 0.25	3.59 ± 0.20	20.09 ± 0.40
C4H::*p*HBMT1-21	2.27 ± 0.05	5.9 ± 0.5	87.6 ± 0.2	5.2 ± 0.1	7.2 ± 0.1	16.34 ± 0.08	3.38 ± 0.23	19.72 ± 0.28
C4H::*p*HBMT1-20	2.46 ± 0.34	5.7 ± 0.4	88.3 ± 0.5	5.2 ± 0.2	6.5 ± 0.3	15.78 ± 0.37	3.39 ± 0.18	19.17 ± 0.54
C4H::*p*HBMT1-9	2.31 ± 0.04	**7.9 ± 0.7**	87.4 ± 0.1	5.2 ± 0.2	7.4 ± 0.1	16.09 ± 0.30	3.43 ± 0.10	19.52 ± 0.35

The S/G ratio and %*p*HB were determined from volume integrations of the signals corresponding to monomeric G, S, and *p*HB units, on a basis where S + G = 100%. By integration of the oxygenated aliphatic region of whole cell walls (CWs) of hybrid poplar, *35S::pHBMT1* and *C4H::pHBMT1*, the major lignin interunit structures were determined (A: β-aryl ether [β-O-4], B: phenylcoumaran [β-5], and C: resinol [β–β]) and are given on an A + B + C = 100% basis. The lignin amount was determined using Klason lignin analysis and expressed as percentage of extractive-free cell wall material. Averages are given ± standard error of the mean (SEM) (*n *=* *3 biological replicates for each line), significant differences compared to the WT are shown in bold (*P *<* *0.05) and were determined via Student’s *t*-test.

To investigate if the increased levels of *p*HB had an effect on the amount of lignin deposited in the cell walls, Klason lignin analysis was also completed. No significant differences were observed between the WT samples and the different *35S::pHBMT1* or *C4H::pHBMT1* transgenic lines (Table 2). Taken together, we clearly see an increase in *p*HB groups in the lignin and/or an increased amount of soluble *p*HB in many of the transgenic lines with no effects on the total cell wall lignin content. These findings demonstrate that *pHBMT1* is indeed involved in the synthesis of monolignol–*p*HB conjugates by coupling *p*-hydroxybenzoyl-CoA with a monolignol.

## Discussion

Although *p*HB esters were first described >65 years ago in poplar (Smith, 1955), the genes/enzymes involved in their biosynthesis have remained unknown until now. In this work, we identified several potential *p*HBMTs using a bioinformatics approach, *in vitro* analysis of 116 synthesized BAHDs, and correlation analyses between *p*HB concentrations and gene expression patterns in unrelated field-grown *P. trichocarpa* genotypes. From the *in vitro* activity assay, only five enzymes displayed positive activity toward *p*-hydroxybenzoyl-CoA. Four of these enzymes were nonselective and displayed *in vitro* activity toward several acyl donor substrates, including acetyl-CoA, benzoyl-CoA, *p*-coumaroyl-CoA, and feruloyl-CoA. Although this does not exclude these four enzymes from being genuine *p*HBMTs, the *in planta* effects could be more complicated as the products that are synthesized by BAHD acyltransferases are largely dependent upon substrate availability (D’Auria, 2006). In addition, two of these corresponding genes were not expressed in the xylem. The most selective enzyme, the one that showed *in vitro* activity only towards *p*-hydroxybenzoyl-CoA and exhibited gene expression in developing xylem, showed a significant positive correlation between gene expression and the amount of cell-wall-bound *p*HB in the different naturally occurring accessions of *P. trichocarpa* that were field grown. A synthetic gene (Potri.001G448000, *pHBMT1*), was therefore transformed into WT poplar, and the ensuing transgenic trees were analyzed for the amount of *p*HB incorporated in the lignin. Recently, a separate study has also reported on the characterization of this same *pHBMT* gene in poplar (Zhao et al., 2021), and observed similar functionality.

Alkaline hydrolysis of phenolics clearly indicated that there was an increase in soluble and cell-wall-bound *p*HB in both *35S::pHBMT1* and *C4H::pHBMT1* transgenic lines. Both DFRC and NMR confirmed that there was an increase in the *p*HB in the lignin of both *35S::pHBMT1* and *C4H::pHBMT1* transgenic poplars. The relative increase in soluble *p*HB was much more apparent than the cell-wall-bound *p*HB. This may be partly associated with the specificity of the secondary cell-wall-specific C4H promoter, rather than the ubiquitous nature of the viral 35S promoter. A possible explanation could be that the *p*HBA in these lines was ester-bound to other unidentified metabolites and thus not to a monolignol and therefore not compatible with lignification and not incorporated into the cell wall. Another plausible hypothesis, and the most likely, is that *p*HBA was conjugated with sinapyl alcohol in these lines, but that the excess S-*p*HB was “detoxified” via glycosylation, transported to the vacuole, and thus not incorporated in the lignin polymer (Le Roy et al., 2016). The latter hypothesis is further supported by the observation that the lines that have increased incorporation of *p*HB in lignin (*35S::pHBMT1* lines 15 and 18, and *C4H::pHBMT1* line 9, determined via NMR and DFRC) are not the lines with the highest expression of the exogenous *pHBMT1* gene. As such, these lines may produce increased levels of monolignol–*p*HB conjugates, but at levels low enough not to stimulate detoxification mechanisms, and thus the conjugates are available to be exported and participate in lignin polymerization rather than being glycosylated and sequestered in the vacuole. It may be possible to further engineer the levels of *p*HB by further increasing the availability of the *p*HBA precursor.

The biological function of lignin acylation remains unknown. One of possible functions could be to increase lignin polymerization rates (Takahama et al., 1996; Goacher et al., 2021). As *p*HB units prefer radical transfer over radical coupling, it could aid in creating S-*p*HB radicals (on the S moiety) since sinapyl alcohol itself (or its conjugate) may not be efficiently oxidized (Ralph et al., 2004; Hatfield et al., 2008; Marjamaa et al., 2009). Hence, *p*HB conjugates may act to increase lignin polymerization. This hypothesis is supported by the fact that *p*HB groups are primarily acylated with S units in poplar, that *p*HB groups are found predominately in the S-rich lignin of fibre in poplar (Goacher et al., 2021), and that *pHBMT1* favors sinapyl alcohol *in vitro* over coniferyl alcohol, as we report here. As lignin is an important response to stresses (Miedes et al., 2014; Cesarino, 2019), it could be that acylated lignin may play a role in plant stress responses, although the evidence for this remains limited. However, poplars originating from more northern latitudes had higher mortality, grew slower, and were more susceptible to *Valsa* and *Melampsora* pathogens (Xie et al., 2009). Some of these same genotypes were also analyzed for cell-wall-bound *p*HB in this study, and we found that the more northern genotypes also displayed some of the lowest levels of cell-wall-bound *p*HB, whereas the more southern genotypes, which had lower mortality and were less susceptible to *Valsa* and *Melampsora* infection, had higher levels of cell-wall-bound *p*HB (Spearman correlation, R_s_: −0.220 and *P=* 0.000128; MacKay, 2019).

For decades, researchers have been attempting to modify the composition of lignocellulosic biomass to improve industrial processing efficiencies (Chanoca et al., 2019). Of primary interest has been the lignin, as biomass recalcitrance, regardless of target end-use (e.g. pulp and paper, bio-ethanol, and/or specialty chemicals) has largely been attributed to the presence of lignin and its association with other cell wall polymers (Mansfield et al., 1999; Fu et al., 2011; Holwerda et al., 2019; Mahon and Mansfield, 2019). As such, lignin quantity and composition has been altered in bioenergy crops/plants by targeted engineering and breeding strategies to improve fodder digestibility, to improve the processing efficiency for the pulp and paper industry, and to lower the cost of the extraction of structural polysaccharides for downstream processing in the production of biofuels (Chanoca et al., 2019). Despite these successes, and even after optimization of the lignin amount and composition, the economic feasibility of using bioenergy crops remains low (Mahon and Mansfield, 2019) as economic hurdles remain without a use for the lignin fraction, which typically comprises 20%–30% of the total biomass. As an alternative to burning the lignin waste stream, current efforts are therefore focused on “lignin-first” bio-refining principles, in which lignin is used for the production of high-value chemicals (Schutyser et al., 2018; Yang et al., 2019; de Vries et al., 2021). An example of such a product is *p*HBA, which can be esterified with various alcohols to form parabens that are widely used as preservatives in the cosmetics and pharmaceutical industries (Yang et al., 2018). In addition, carboxylation of *p*HBA can be used to make terephthalate, the key precursor in PET plastics (Bai et al., 2016), which is currently produced from the petrochemical *p*-xylene. Recently, it was also shown that acetaminophen can be made from *p*HBA (Ralph et al., 2019). Currently, *p*HBA is produced via the Kolbe–Schmitt reaction from carbon dioxide and potassium phenoxide, which itself is derived from petrochemicals (Ritzer and Sundermann, 2000). In the future, *p*HBA could therefore be a high-value coproduct from the lignin-first bio-refinery in which, like *p*-coumarate (Karlen et al., 2020; Timokhin et al., 2020), it is easily clipped-off via a hydrolysis reaction from an engineered lignin polymer, rendering the lignocellulosic biomass of more value, and ultimately improving the economics of alternative energy from renewable biomass.

## Materials and methods

### Alkaline hydrolysis of cell-wall-bound phenolics

Tissue collection for the evaluation of the diversity of *p*HB from 316 unrelated *P.* *trichocarpa* genotypes was performed as described previously (Porth et al., 2013a, 2013b). Dried and ground xylem tissue was first subjected to an acetone extraction for 24 h using a Soxhlet apparatus. Extractive-free wood tissue (30 mg) was then weighed into vials as triplicate samples. Cell-wall-bound *p*HB was determined as previously described (Goacher et al., 2021).

### Identification of candidate BAHDs from *P. trichocarpa* and gene synthesis

The entire complement of protein sequences from *P. trichocarpa* (genome ID: 210, v3.0) was obtained from Phytozome and matched against the HMM profile for the Pfam02458 family of CoA-dependent transferases to identify 147 proteins of interest. Proteins were examined for completeness, where sequences that were too short to make a complete PF02458 protein were excluded from further analysis (resulting in 127 proteins of interest). Six sequences were rejected as redundant and essentially identical to an included sequence (resulting in 121 proteins of interest). After a ClustalW alignment, a phylogenetic tree was created in MEGA X (Kumar et al., 2018; Stecher et al., 2020) using the maximum likelihood method and JTT matrix-based model (default settings), with a bootstrap of 1,000 (Jones et al., 1992).

The sequences were then tested for a number of characteristics that are known to lead to poor performance in recombinant protein expression systems. Specifically, they were evaluated for the presence of signal peptides, transmembrane domains, and low-complexity regions. No sequences contained signal peptides or possessed extensive low-complexity regions. Putative transferases were matched with their nucleotide sequences, and sent for synthesis at the US Department of Energy Joint Genome Institute. As many of the known acyltransferase genes are GC-rich (>60%) and therefore potentially problematic for DNA amplification and recombinant protein expression, the genes were codon-altered for ease of gene synthesis. The gene synthesis platform produced open reading frames in the wheatgerm cell-free expression vector pEU (Takai et al., 2010). No synthesis product was obtained for five genes, yielding 116 BAHDs successfully synthesized for subsequent analysis (Supplemental Data Set S1).

### Co-expression analysis

Co-expression analysis of the potential *p*HBMT clade with lignin genes was performed via the exNet tool on www.popgenie.org (Sjödin et al., 2009; Sundell et al., 2015), in which the AspWood database was used for the analysis (Sundell et al., 2017); the layout employed was set to “Cose-Bilkent” and a threshold of ≥3 was used.

### Gene expression in *P. trichocarpa* collection

Xylem scrapings were collected from 195 unrelated, 4-year-old *P. trichocarpa* genotypes that were grown in a common garden, as described previously (McKown et al., 2014). RNA was isolated from xylem scrapings, purified, and quantified prior to RNA-seq library preparation and sequencing in an Illumina HiSeq. 2000. The RNA-seq data were analyzed as previously described (Hefer et al., 2015; Ribeiro et al., 2020).

The amount of *p*HBA was correlated with the expression of the candidate BAHD genes. For this, 164 unrelated *P. trichocarpa* genotypes could be used, as these genotypes are genetically identical (scions) and were grown in both common gardens.

### *In vitro* activity screening of the synthetic *p*HBMTs

Methods and rational for our implementation of cell-free protein translation are previously described (Takasuka et al., 2014; Makino et al., 2014). Messenger RNA was prepared by adding 1.6 U of SP6 RNA polymerase and 1 U RNase inhibitor (Promega Corporation, Madison, WI, USA) to plasmid DNA solutions containing sub-cloned pEU7 plasmid DNA, 2.5 mM of UTP, CTP, ATP, and GTP, 20 mM magnesium acetate, 2 mM spermidine HCl, 10 mM dithiothreitol (DTT), and 80 mM HEPES-KOH. Reactions were incubated at 37°C for 4 h. This mRNA was then employed as the template for cell-free translation using the WEPRO1240 Series Expression Kit (CellFree Sciences, Yokohama, Japan). After translation, the reactions were sampled and evaluated for expression and protein characteristics using sodium dodecyl sulphate–polyacrylamide gel electrophoresis (Supplemental Figure S4). Trp-fluorescence or densitometric quantification of protein bands, migration position, and solubility of each reaction was analyzed to approximate yield, indicate if the protein was properly folded, and determine whether it was a good candidate for scale-up purification.

Translated enzymes were screened for activity with acetyl-CoA, benzoyl-CoA, *p*-hydroxybenzoyl-CoA, *p*-coumaroyl-CoA, and feruloyl-CoA, and all three monolignols (*p*-coumaryl, coniferyl, and sinapyl alcohol). Acetyl-CoA and benzoyl-CoA were purchased (Sigma-Aldrich), whereas feruloyl-CoA, *p*-coumaroyl-CoA, and *p*-hydroxybenzoyl-CoA were enzymatically synthesized using the *Nt4CL1* enzyme as described by Beuerle and Pichersky (2002). Monolignols and monolignol conjugate standards for LC–MS were synthesized as previously described (Zhu et al., 2013). For individual enzyme reactions, the cell-free translation products were added to a reaction mixture containing 50 mM sodium phosphate buffer (pH 6), 1 mM DTT, 1 mM CoA thioester, and 1 mM monolignol mixture containing each monolignol. After a 1 h incubation at room temperature, reactions were stopped with the addition of 100 mM HCl. MeOH was added to each reaction and filtered through 0.2 μm nylon syringe filters and analyzed for product formation using LC–MS. Pooled enzyme reactions were performed by scaling up the reaction to accommodate 10 enzymes, while maintaining the reaction mixture concentrations listed above. Identification of enzymes with positive *in vitro* activity was accomplished using a Shimadzu LC–MS 8040 (Prominence LC linked to a triple-quadrupole mass spectrometer) equipped with a Kinetex 5u XB-C18 column (Phenomenex; 250 mm × 4.6 mm × 5 μm, 100 Å, P/N:00G-4605-E0) held at 50°C. The mobile phase was a binary gradient of water (A) and methanol (B) at a flow rate of 1 mL·min^−1^. The gradient protocol ran as follows: 0 min, 5% B; 2 min 5% B; 30 min, 100% B; 34 min, 100% B; 35 min, 5% B; 45 min 5% B. The products were first analyzed using a PDA detector (scanning from 250 to 400 nm), and then by MS. The eluent was ionized using a DUIS probe head operating in ESI and APCI modes (nebulizing gas 2.5 L·min^−1^, drying gas 15 L·min^−1^) and subjected to a voltage of 4.5 kV to negatively ionize the samples scanning in both positive and negative mode from 120 to 600 *m*/*z*. Enzymes showing positive *in vitro* activity with the production of expected products (with reference to authentic standards) were identified as putative *p*HBMTs and subjected to a further screening with each monolignol acceptor provided independently.

Kinetics reactions were performed by preparing reaction mixtures as described above with 50 mM sodium phosphate buffer (pH 6), 1 mM DTT, 0.375–1.5 mM *p*-hydroxybenzoyl-CoA, and 0.375–1.5 mM of each monolignol alcohol substrate (*p*-coumaryl alcohol, coniferyl alcohol, or sinapyl alcohol), adjusted to 50 µL with deionized H_2_O. The reactions were initiated by adding 0.75 µL of cell-free *p*HBMT enzyme (approximately 410 ng enzyme). Enzyme kinetics were measured by directly injecting 2 µL of the reaction mixture into the LC–MS every 15 min for 90 min. Reactions were run on a triple quadrupole LC–MS (Shimadzu LC–MS 8040) equipped with a Symmetry C18 column (Waters; 4.6 mm × 250 mm × 5 µm) held at 50°C. Mobile phase A, water, and mobile phase B, methanol were used with the following gradient protocol: initial concentration 25% B, followed by a linear gradient to 50% B over 3 min, a further linear gradient to 90% B over 2 min, held at 90% B for 30 s, then decreased to 25% B in a linear gradient over 30 s, and finally held at 25% B for 9 min (15 min total run time). A constant flow rate of 1 mL·min^−1^ was used, and the autosampler temperature was set at 22°C. A standard curve was generated for each monolignol *p*HB product (H-*p*HB, G-*p*HB, and S-*p*HB) using authentic standards, with seven concentrations ranging from 0.0005 mM to 0.2 mM, and multiple reaction monitoring (negative mode) events were created for each product: H-*p*HB 305 > 137 (CE 10), 305 > 93 (CE 25); G-*p*HB 335 > 137 (CE 15), 335 > 93 (CE 30); S-*p*HB 365 > 137 (CE 15), 365 > 93 (CE 30). These standard curves were used to calculate the amount of product formed (mM) at each time point. Kinetic parameters (*V*_max_ and *K*_M_) were calculated by generating a Lineweaver-Burk plot of the reaction rates from each reaction.

### Construct development and poplar transformation

*pHBMT1* was amplified from the pEU7 plasmid containing the synthesized putative *pHBMT1* gene with primer: GGGGACCACTTTGTACAAGAAAGCTGGGTTCACAGACGATGACGAATTGGC and primer: GGGGACAAGTTTGTACAAAAAAGCAGGCTATGCCAACCCCAACCAG, containing *attB* recognition sites for cloning into the Gateway pDONR/Zeo vector (Thermo Fisher Scientific, USA) following the standardised Gateway cloning methods, and transformed into *Escherichia* *coli* One Shot competent cells (Thermo Fisher Scientific, Waltham, MA, USA). The confirmed sequence was then transferred from pDONR into the plant expression vector pK7WG2 using Gateway LR Clonase according to the manufacturer’s instructions, and was then transformed into *E. coli*. The final construct, henceforth referred to as *35S::pHBMT1*, was then transformed into *Agrobacterium tumefaciens* strain EHA105 for poplar transformations.

For the *C4Hp::pHBMT1* constructs, the Arabidopsis (*Arabidopsis thaliana*) *AtC4H* promoter (*AtC4Hp*) was amplified from the *pTkan-pC4H::schl::qsuB* plasmid (Eudes et al., 2015), using primers: GCTCTAGAGCGGCCGCCTGCAGGTCGACCTAGGGGGCGAGAGTAATTG, containing an XbaI restriction enzyme site and GTGAGCTCTCCCATATGGTCGACGGAATGAGAGACGAGAGC, containing a SacI restriction enzyme site (pA6pC4HsQsuB plasmid from Eudes et al., 2015). The purified *AtC4Hp* PCR product and the plant overexpression vector pK7WG2 were subjected to SacI+XbaI or SacI+SpeI restriction enzyme digestions, respectively. Fragments of these digestions were separated by agarose gel-electrophoresis, purified, and then ligated together with T4 DNA ligase (Thermo Fisher Scientific) to create the *AtC4Hp-pK7WG2*. Gateway LR Clonase was used in the same way as above to transfer the confirmed *pHMBT1* sequence from pDONR into *AtC4Hp-pH7WG2*. The final construct, henceforth referred to as *C4H::pHBMT1*, was transformed into *A. tumefaciens* strain EHA105.

Transformation, growth, and selection of transgenic P39 hybrid poplar was performed as described previously (Wilkerson et al., 2014).

### Plant material

Once at least eight plants of each of the three highest expressing lines were available, 6-week-old plantlets were transferred to two-gallon pots containing perennial mix (Westcreek Farms Ltd., Fort Langley, BC, Canada) in the University of British Columbia horticultural greenhouse. To minimize mortality, high humidity conditions were maintained by covering plants with clear plastic cups and misting each plant twice a week with distilled water for 2 weeks. Plants were grown with 18 h of light provided by a mix of deep red/white to low blue lights, and deep red/white to medium blue lights at 20°C–26°C, and watered with fertilized water four times per week. After 4 months of growth, the stem diameter was measured 10 cm above the root collar, and the height of the tree was recorded from the apex of the tree to the soil level. Stem tissue was harvested by cutting 10 cm above root collar and removing the top to yield 100 cm of stem. Xylem scrapings were collected from debarked stems and flash-frozen in liquid nitrogen at −80°C for RT-qPCR analysis. The bottom 15 cm of the debarked stems were also selectively harvested and placed at 50°C to dry for 72 h for cell-wall compositional analysis. After that, the pith was removed and stems were cut into smaller matchstick-sized pieces. This xylem was then ground in a Wiley Mini-mill (Thomas Scientific) to pass a 40-mesh (0.4 mm) sieve. Both *C4Hp::pHBMT1* and *35S::pHBMT1* P39 transformations were grown with a minimum of eight biological replicates, with three replicates being harvested and used for experiments. All lines were grown in parallel with WT P39 trees.

### Expression analysis

RT-qPCR techniques were employed to determine the relative transcript levels in the developing xylem tissue of 4-month-old poplar overexpression lines. RNA isolation was performed using the TRIzol Reagent protocol (Thermo Fisher Scientific) with the addition of a second EtOH wash using 95% EtOH. To remove contaminating DNA, isolated RNA was subjected to TURBO DNase treatment following the manufacturer’s instructions (Thermo Fisher Scientific). cDNA was synthesized by using the EasyScript Plus cDNA Synthesis Kit according to manufacturer’s instructions (Applied Biological Materials, Vancouver, BC, Canada). This cDNA was then used as the template in RT-qPCR reactions using BrightGreen Express 2X qPCR MasterMix (Applied Biological Materials) and a Bio-Rad CFX96 Touch Real-Time PCR Detection System (Bio-Rad Laboratories, Hercules, CA, USA) according the manufacturer’s instructions. cDNA from all transgenic lines and WT trees was pooled and serially diluted five times to determine primer efficiency and an appropriate template concentration. Gene-specific primers used in RT-qPCR reactions were: ACATTCGTAGTCTGGCCGAT and ACCCCAACCGTAATCCACTT, and reference gene primers were: GGCATTAAGTTTTGTCGGTCTG and GCGGTTCATCATTTCATCTGG for *PtEF1*β amplification. Relative gene expression levels were determined and normalized to the highest expressing line (no expression of the synthetic *pHBMT1* gene was detected in WT trees).

### Analysis of methanol-soluble *p*HBA in xylem

Dried stem tissue was weighed out in triplicate for each transgenic line and subjected to a MeOH extraction for determination of phenolics. Extraction was achieved by adding methanol/water/HCl (48.5:48.5:1) solution and incubating samples at 50°C for 4 h. Extracted samples were then pelleted by centrifugation The supernatant was retained and divided into two 500 μL aliquots. To liberate ester-bound phenolics, one aliquot was subjected to base-mediated saponification using 0.2 M NaOH, and incubated at 30°C at 500 rpm for 24 h. The reactions were then stopped with the addition of 72% (w/w) H_2_SO_4_. The ether phase of these samples was then isolated by ethyl ether phase separation. Nano-pure water and ethyl ether were added to both the saponified and nonsaponified samples, briefly vortexed, and left to separate. The upper phase was retained and subjected to a second ethyl ether phase separation. The upper phase was again retained and pooled with the first phase separation, concentrated using a 5301 Vacufuge at 1,400 rpm for 15 min. Pellets were resuspended in 1 mL MeOH and analyzed via HPLC as previously described (Goacher et al., 2021).

### Derivatization followed by reductive cleavage

Incorporation of *p*HB conjugates into lignin was determined via the DFRC procedure (which cleaves ether bonds, but leaves ester bonds intact), as previously described (Karlen et al., 2016; Regner et al., 2018).

### Nuclear magnetic resonance

After preparation of ball-milled cell wall material, enzyme lignin was isolated and 2D heteronuclear single-quantum coherence NMR spectra were acquired as previously described (Kim and Ralph, 2010; Mansfield et al., 2012; Kim et al., 2017).

### Klason lignin content

Total lignin content was determined using a modified Klason lignin analysis as previously described (Huntley et al., 2003).

### Statistical analysis

Statistical analysis was performed using R version 1.4.1106 (RStudio Inc., Boston, MA, USA) and SPSS version 27 (IBM). Spearman correlation analyses between *p*HB content and gene expression data were conducted using the statistical packages car v.2.1-4 (Fox and Weisberg, 2011) and an adjusted significance level was determined using the Bonferroni correction. Spearman’s correlation analysis between *p*HB content and latitude was conducted in SPSS. Significant differences between *35S::pHBMT1* or *C4H::pHBMT1* lines and P39 (WT) were determined using Student’s *t*-test (*p *<* *0.05).

### Accession numbers

*pHBMT1*: Potri.001G448000. Putative *pHBMTs*: Potri.001G447300, Potri.001G447400, Potri.001G447500, Potri.011G153500.

## Supplemental data

The following materials are available in the online version of this article.

**Supplemental Data Set S1.** Bioinformatic identification of putative *p*HBMTs from *P. trichocarpa.*

**Supplemental Table S1.** An overview of the phenylpropanoid genes used in the co-expression network analysis (Supplemental Figure S2).

**Supplemental Figure S1.** A maximum likelihood phylogenetic tree of identified BAHD ATs from *P. trichocarpa.*

**Supplemental Figure S2.** Co-expression network of lignin biosynthetic genes with putative *pHBMTs*.

**Supplemental Figure S3.** LC–MS spectra of the enzyme kinetic reactions.

**Supplemental Figure S4.** SDS–PAGE analysis of the solubility of BADH transferases investigated in this manuscript.

## Supplementary Material

kiab546_Supplementary_Data

## Notes

L.d.V., H.A.M., R.A.S., Y.M., S.D.K., F.U., E.M., C.B., K.V., E.T.B. designed the experiments, performed the experiments, and analyzed the data. B.G.F., J.R., and S.D.M. supervised the experiments. L.d.V. wrote the article in collaboration with H.A.M., and of all other authors. S.D.M. supervised and completed the writing and agrees to serve as the author responsible for contact and communication.

The author responsible for distribution of materials integral to the findings presented in this article in accordance with the policy described in the Instructions for Authors (https://academic.oup.com/plphys/pages/general-instructions) is Shawn D. Mansfield: shawn.mansfield@ubc.ca.
