# Supplementary material for: pHBMT1, a BAHD-family monolignol acyltransferase, mediates lignin acylation in poplar
Source: Plant Physiol. 2021 Nov 22;188(2):1014–27. doi: 10.1093/plphys/kiab546 (PMC8825253; doi:10.1093/plphys/kiab546)
Supplement: kiab546_Supplementary_Data [file kiab546_supplementary_data.docx]

***p*HBMT1, a BAHD-family monolignol acyltransferase, mediates lignin acylation in poplar**

Lisanne de Vries*^1,2^, Heather A. MacKay*^1^, Rebecca A. Smith^2,3^, Yaseen Mottiar^1,2^, Steven D. Karlen^2,3^, Faride Unda^1,2^, Emilia Muirragui^1^, Craig Bingman^3^, Kirk Vander Meulen^3^, Emily T. Beebe^3^, Brian G. Fox^3^, John Ralph^2,3^, Shawn D. Mansfield^1,2^

*These authors contributed equally to this work

Corresponding author: Shawn D. Mansfield, [shawn.mansfield@ubc.ca](mailto:shawn.mansfield@ubc.ca)

^1^ Department of Wood Science, Faculty of Forestry, University of British Columbia, Vancouver, BC, V6T 1Z4, Canada.

^2^ US Department of Energy (DOE) Great Lakes Bioenergy Research Center, the Wisconsin Energy Institute, University of Wisconsin ‐ Madison, Madison, WI 53726, USA.

^3^ Department of Biochemistry, University of Wisconsin-Madison, 433 Babcock Drive, Madison, WI, 53706, USA

**Supplemental Dataset S1: ­Bioinformatic identification of putative *p*HBMTs from *P. trichocarpa***

The following 20 sequences were too short to make a complete PF02458 protein and so were dropped from further evaluation:

Potri.001G387200 92 aa

Potri.001G395600 159 aa

Potri.003G082100   223 aa

Potri.004G017900   265 aa

Potri.004G096200 237 aa

Potri.005G250400    59 aa

Potri.007G140000    71 aa

Potri.007G140100   191 aa

Potri.010G054200   171 aa

Potri.010G054000   274 aa

Potri.012G126200   112 aa

Potri.013G072500   180 aa

Potri.013G074600   193 aa

Potri.014G059900    84 aa

Potri.017G010000    73 aa

Potri.017G049400   186 aa

Potri.017G049900    82 aa

Potri.018G021200   146 aa

Potri.019G001300   141 aa

Potri.T112100 231 aa

The following six pairs of sequences were near identical, so only one of each pair was submitted to JGI for gene synthesis:

Potri.003G057100 and Potri.003G057200; Potri.003G057100 was synthesized

Potri.011G124200 and Potri.011G124300; Potri.011G124200 was synthesized

Potri.T019300 and Potri.T019400; Potri.T019300 was synthesized

Potri.004G110400 and Potri.004G110700; Potri.004G110400 was synthesized

Potri.004G103200 and Potri.004G103300; Potri.004G103200 was synthesized

Potri.006G157100 and Potri.006G158400; Potri.006G157100 was synthesized

The following diagram shows the layout of 121 synthesized genes received from JGI. Wells marked in orange (5) failed to yield a plasmid with the proper gene sequence as so were dropped from further experimentation. The remaining 116 genes were subjected to the cell-free protein synthesis protocol and individual translation reactions were combined to carry out multiplexed functional assays. Genes marked in blue were active

|  | 1 | 2 | 3 | 4 | 5 | 6 | 7 | 8 | 9 | 10 | 11 | 12 |
| --- | --- | --- | --- | --- | --- | --- | --- | --- | --- | --- | --- | --- |
| A | Potri.006G034100 | Potri.007  G143500 | Potri.005G028400 | Potri.001G447900 | Potri.008G034400 | Potri.010G056400 | Potri.004G096400 | Potri.005G250500 | Potri.001G152500 | Potri.007G139400 | Potri.010G053900 | Potri.010G053800 |
| B | Potri.018  G032700 | Potri.017  G068500 | Potri.006G010200 | Potri.005G028500 | Potri.008G034300 | Potri.014G113600 | Potri.005G230900 | Potri.018G105500 | Potri.015G126600 | Potri. T112000 | Potri. T111900 | Potri.017G011600 |
| C | Potri.017  G010600 | Potri.001  G042900 | Potri.015G100800 | Potri. T178300 | Potri.006G010300 | Potri.005G028200 | Potri.004G017600 | Potri.015G127000 | Potri.010G186300 | Potri.008G071200 | Potri.019G001400 | Potri.005G153600 |
| D | Potri. T124500 | Potri.017  G029300 | Potri.018G105400 | Potri.006G036100 | Potri.008G180400 | Potri.013G074300 | Potri.018G104800 | Potri.018G104700 | Potri.014G166600 | Potri.003G057100 | Potri.019G001200 | Potri.002G244600 |
| E | Potri.010G056300 | Potri.017  G024800 | Potri.001G326300 | Potri.001G455200 | Potri.001G319200 | Potri.005G028000 | Potri.004G096100 | Potri.013G112100 | Potri.005G028100 | Potri.015G147300 | Potri.010G054100 | Potri.008G033500 |
| F | Potri.008G034100 | Potri.004G053500 | Potri.001G128100 | Potri.001G447800 | Potri.002G010700 | Potri.006G165200 | Potri.013G039900 | Potri.013G039700 | Potri.005G052200 | Potri.019G003000 | Potri.010G180000 | Potri.011G124500 |
| G | Potri.011G124200 | Potri.019G002900 | Potri.002G032200 | Potri.003G019900 | Potri.018G109900 | Potri.014G025600 | Potri.008G065000 | Potri.008G034200 | Potri.T131600 | Potri.013G074400 | Potri.003G183900 | Potri.013G074500 |
| H | Potri.001G127600 | Potri.019G043600 | Potri.014G025500 | Potri.001G310000 | Potri.001G127400 | Potri.004G093800 | Potri.011G153500 | Potri.001G447500 | Potri.001G447400 | Potri.001G447300 | Potri.001G447700 | Potri.019G118000 |
|  |  |  |  |  |  |  |  |  |  |  |  |  |
|  | 1 | 2 | 3 | 4 | 5 | 6 | 7 | 8 | 9 | 10 | 11 | 12 |
| A | Potri.001  G448000 | Potri.006G157100 | Potri.004G120700 | Potri.004G103200 | Potri.004G103100 | Potri.009G063500 | Potri. T018800 | Potri.017G094700 | Potri.004G096300 | Potri.006G097500 | Potri. T019300 | Potri.004G110400 |
| B | Potri.004  G109300 | Potri.001G395400 | Potri.010G208100 | Potri.001G395700 | Potri.009G019700 | Potri.019G126400 | Potri.010G192400 | Potri. T018300 | Potri.007G003800 | Potri.012G144500 | Potri.001G395900 | Potri.003G057000 |
| C | Potri.016  G112400 |  |  |  |  |  |  |  |  |  |  |  |
| D |  |  |  |  |  |  |  |  |  |  |  |  |
| E |  |  |  |  |  |  |  |  |  |  |  |  |
| F |  |  |  |  |  |  |  |  |  |  |  |  |
| G |  |  |  |  |  |  |  |  |  |  |  |  |
| H |  |  |  |  |  |  |  |  |  |  |  |  |

**Supplemental Table S1: An overview of the phenylpropanoid genes used in the co-expression network analysis (Supplemental Figure S2).** Genes that are co-expressed with *pHBMT1* are shown in bold. *4CL*, *4-Coumarate:CoA Ligase*; *C3H*, *p-Coumarate 3-Hydroxylase*; *C4H*, *Cinnamate 4-Hydroxylase*; *CAD*, *Cinnamyl Alcohol Dehydrogenase*; *CCoAOMT*, *Caffeoyl-CoA O-Methyltransferase*; *CCR*, *Cinnamoyl-CoA Reductase*; *COMT*, *Caffeic Acid O-Methyltransferase*; *CSE*, *Caffeoyl Shikimate Esterase*; *F5H*, *Ferulate 5-Hydroxylase*; *HCT*, *Hydroxycinnamoyl-CoA Shikimate/Quinate Hydroxycinnamoyl Transferase*; *PAL*, *Phenylalanine Ammonia-Lyase*.

| **Phenylpropanoid gene** | **Gene identification number** |
| --- | --- |
| *PAL* | **Potri.006G126800**, **Potri.008G038200**, **Potri.010G224100**, **Potri.010G224200**, **Potri.016G091100** |
| *C4H* | Potri.006G078100, **Potri.013G157900**, **Potri.018G146100**, **Potri.019G130700** |
| *C3H* | Potri.006G033300, Potri.016G031000 |
| *4CL* | **Potri.001G036900**, Potri.003G099700, **Potri.003G188500**, Potri.004G102000, Potri.005G248500, **Potri.006G169600**, Potri.010G057000, Potri.019G049500 |
| *HCT* | Potri.001G042900, **Potri.003G183900**, Potri.018G104700, Potri.018G104800 |
| *CSE* | **Potri.001G175000**, **Potri.003G059200** |
| *CCoAOMT* | **Potri.001G304800**, Potri.008G136600, Potri.008G136700, Potri.009G099800 |
| *CCR* | **Potri.001G045500**, Potri.001G046100, **Potri.003G181400**, Potri.004G230900, Potri.009G076300 |
| *F5H* | **Potri.005G117500**, **Potri.007G016400** |
| *COMT* | Potri.001G451100, Potri.002G076800, Potri.011G150500, **Potri.012G006400**, Potri.014G106500, Potri.014G106600, Potri.015G003100, Potri.016G101600 |
| *CAD* | Potri.001G268600, Potri.001G300000, Potri.001G307200, Potri.002G018300, Potri.005G243700, Potri.009G062800, Potri.009G063100, Potri.009G063300, Potri.009G063400, **Potri.009G095800**, Potri.011G148100, Potri.011G148200, Potri.016G078300 |


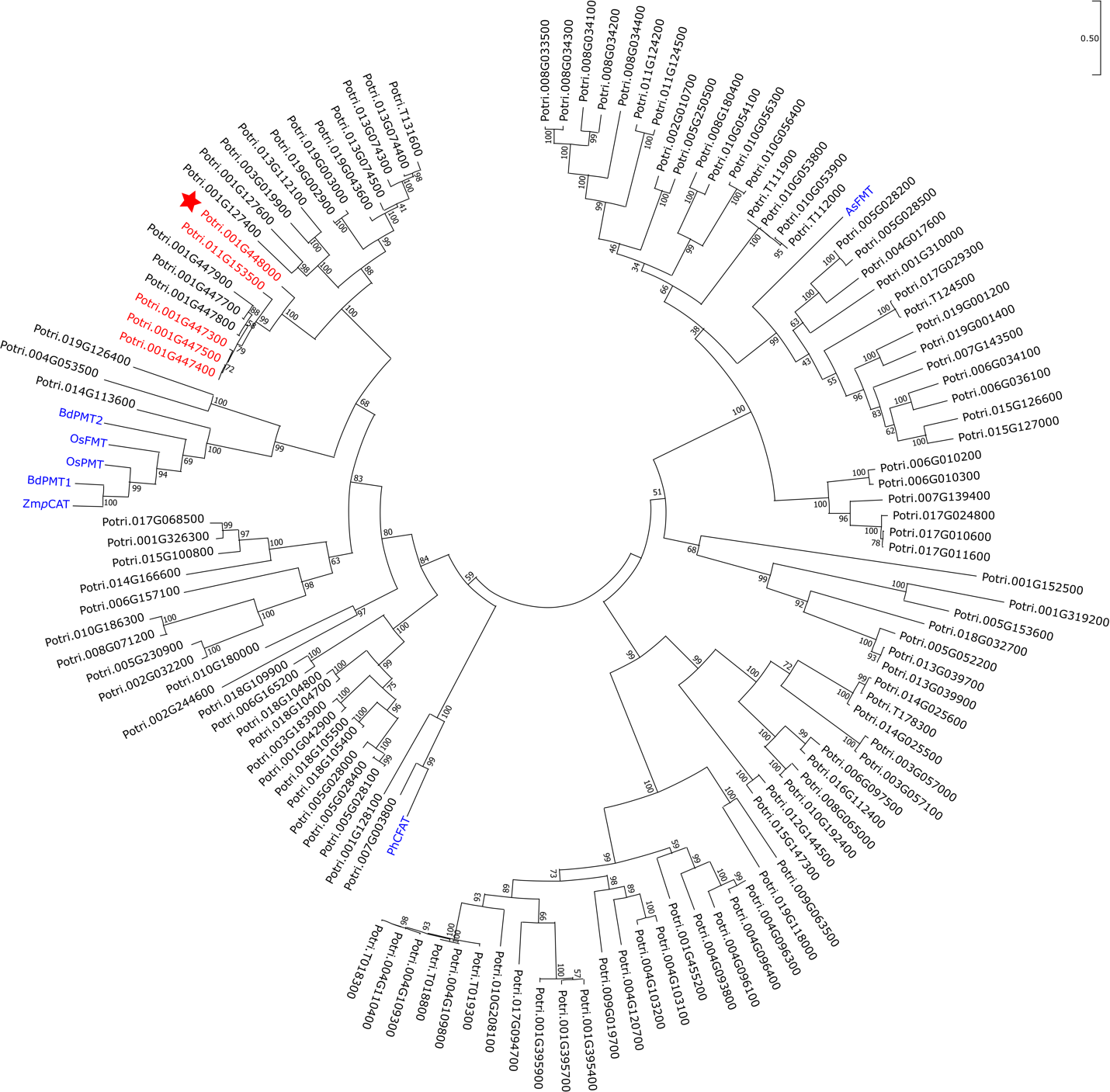


**Supplemental Figure S1: A Maximum likelihood phylogenetic tree of identified BAHD ATs from *P. trichocarpa.*** The 5 BAHD ATs that showed *in vitro* activity with *p*-hydroxybenzoyl-CoA cluster together in a clade made-up of 8 BAHD ATs, indicated in red. The putative *p*HBMT that is further investigated in this manuscript is indicated with a star. The tree is drawn to scale, with branch lengths measured in the number of substitutions per site. Other identified BAHD ATs that use monolignols as substrate are indicated in blue; BdPMT1/2: *Brachypodium distachyon p*-Coumaroyl-CoA Monolignol Transferase 1/2 (Bradi2g36910.1/Bradi1g36980.1), OsFMT: *Oryza* *sativa* Feruloyl-CoA Monolignol Transferase Os05g19910 (LOC_Os05g19910), OsPMT: *Oryza* *sativa p*-Coumaroyl-CoA Monolignol Transferase (LOC_Os01g18744.1), Zm*p*CAT: *Zea mays p*-Coumaroyl-CoA Hydroxycinnamyl Alcohol Transferase (BT042717.1), PhCFAT: *Petunia* × *hybrida* Coniferyl Alcohol Acetyltransferase (ABG75942), AsFMT: *Angelica sinensis* Ferulate Monolignol Transferase (AHL24755). Bootstraps values are shown next to the branches (1000 replicates). The analysis was conducted in MEGA X.


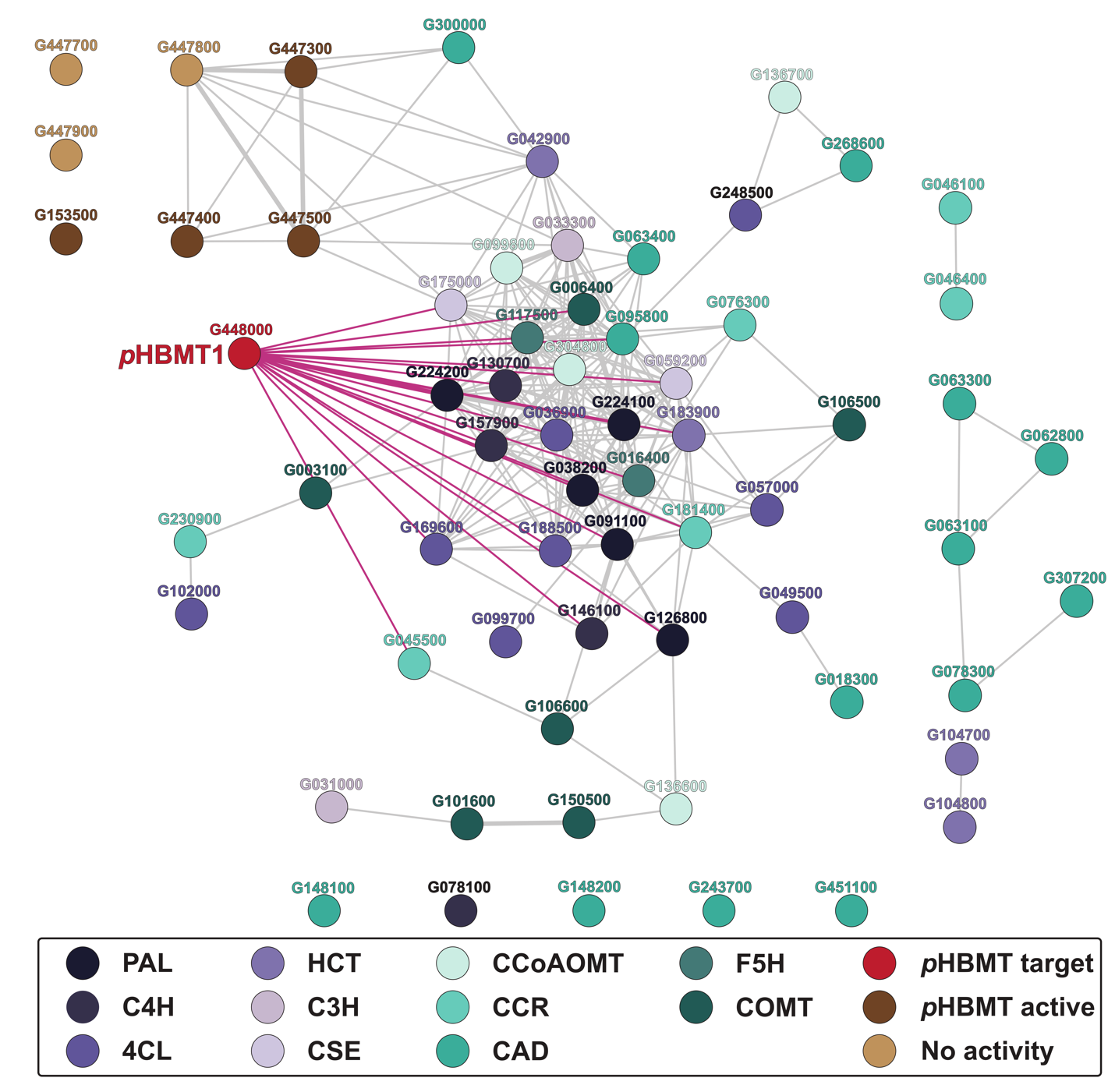


**Supplemental Figure S2: Co-expression network of lignin biosynthetic genes with putative *p*HBMTs.** The network was made with the aid of [www.popgenie.org](http://www.popgenie.org), using the exNet tool, with the AspWood database, Cose-Bilkent lay-out and a threshold >=3 (Sjödin et al., 2009; Sundell et al., 2015).


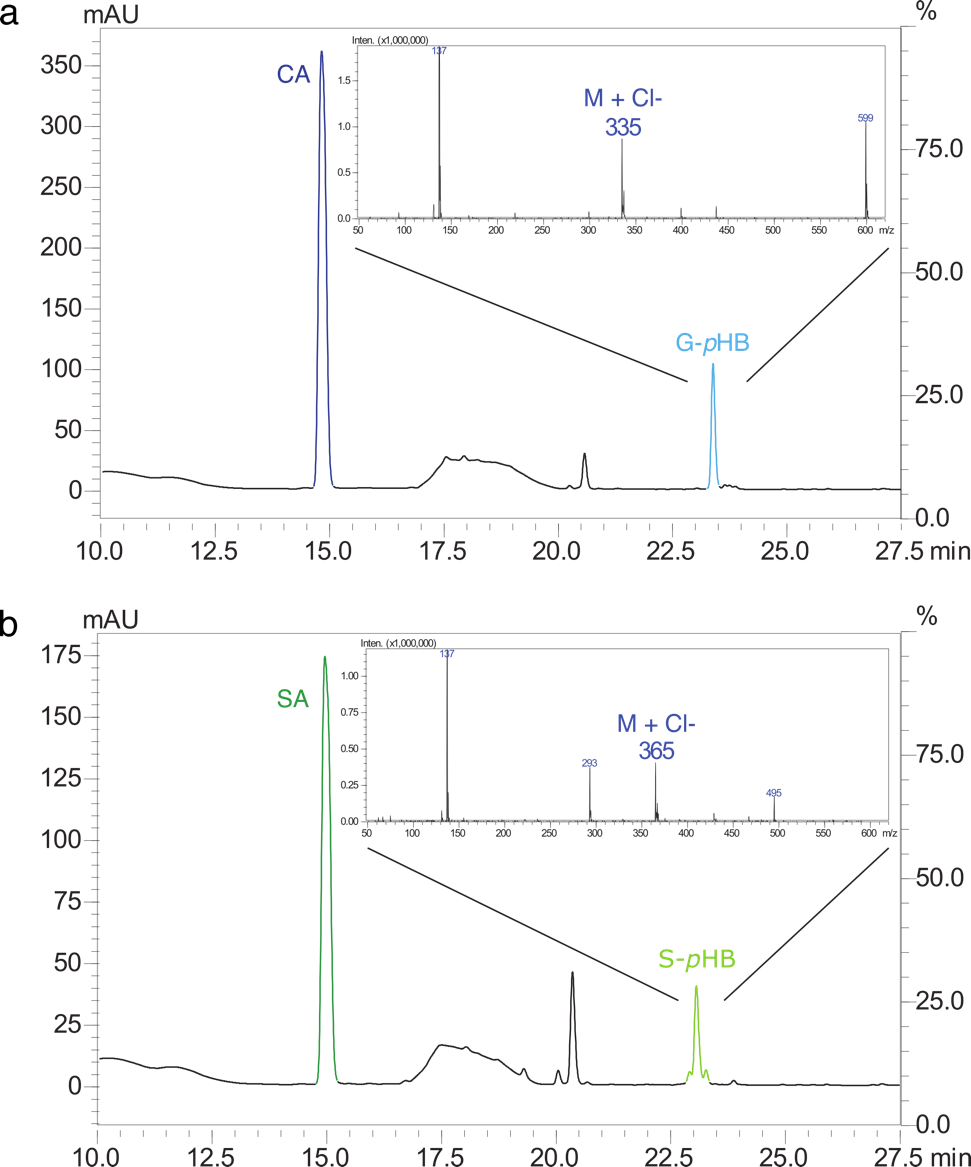


**Supplemental Figure S3: LC-MS spectra of the enzyme kinetic reactions.** (a) Coniferyl alcohol (CA) and *p*-hydroxybenzoyl-CoA resulting in the formation of G-*p*HB. (b) Sinapyl alcohol (SA) and *p*-hydroxybenzoyl-CoA resulting in the formation of S-*p*HB. G-*p*HB 335>137 (CE 15), 335>93 (CE 30); S-*p*HB 365>137 (CE 15), 365>93 (CE 30).

**
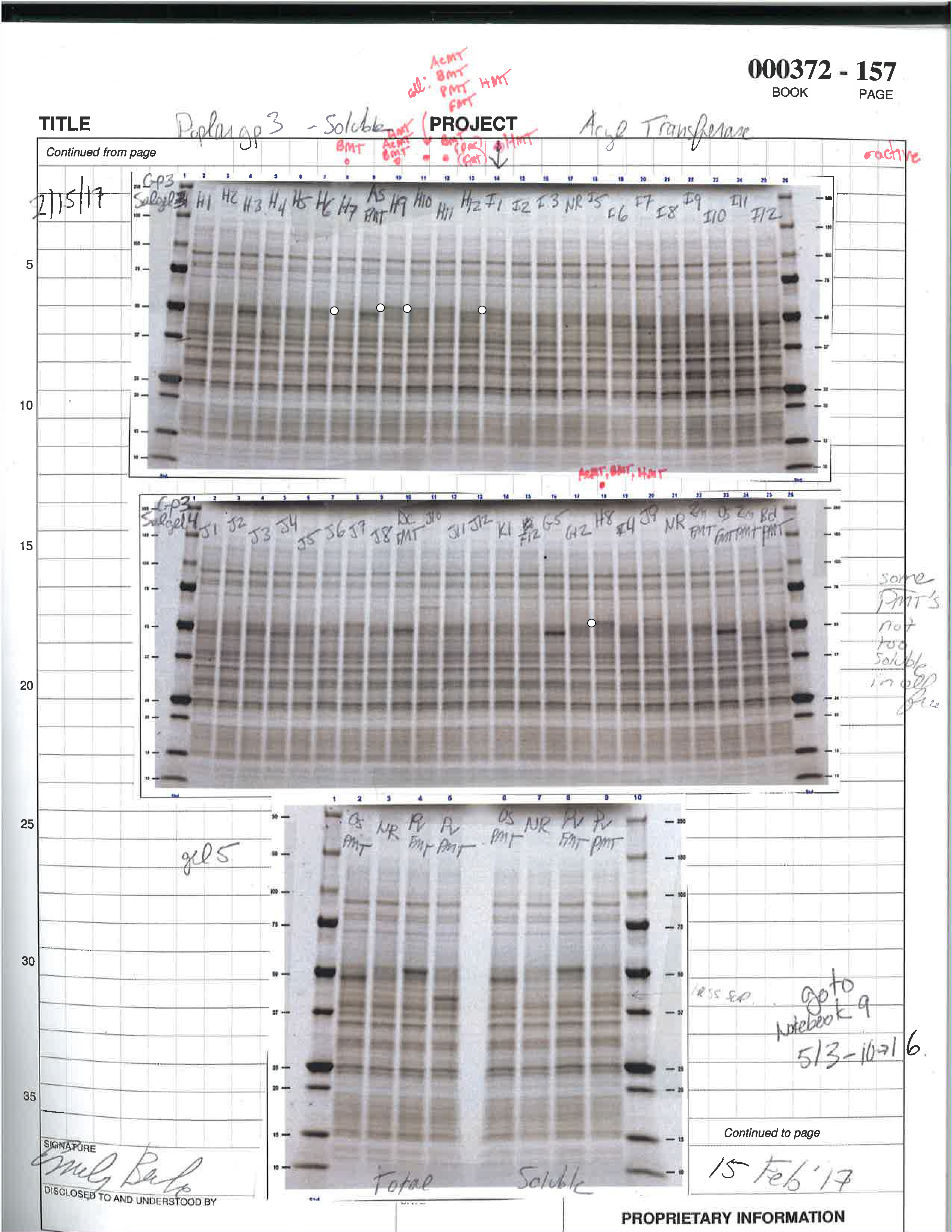
**

**Supplemental Figure S4:** **SDS-PAGE analysis of the solubility of BADH transferases investigated in this manuscript**. Subsequent annotations of detected catalytic activities are in red. A Copy of page 157 from lab notebook 000372. Lanes and expressed proteins are identified by a white circle to the left of the assigned protein band: H7, Potri.011G153500; H9, Potri.001G447400; H10, Potri.001G447300; I1, Potri.001G448000, H8, Potri.001G447500. NR, no reaction is a wheatgerm cell extract control lane.
